# The Expression of the Hepatocyte SLAMF3 (CD229) Receptor Enhances the Hepatitis C Virus Infection

**DOI:** 10.1371/journal.pone.0099601

**Published:** 2014-06-13

**Authors:** Flora Cartier, Ingrid Marcq, Florian Douam, Christèle Ossart, Aline Regnier, Véronique Debuysscher, Dimitri Lavillette, Hicham Bouhlal

**Affiliations:** 1 EA 4666, UFR de Médecine, CAP-Santé (FED 4231), Université de Picardie Jules Verne, Amiens, France; 2 INSERM U1053, Laboratoire de Physiologie du Cancer du Foie, Université Bordeaux Segalen, Bordeaux, France; 3 UMR CNRS 5557 Ecologie Microbienne, Université Lyon 1, Villeurbanne, France; 4 Laboratoire de Thérapie Cellulaire, CHU Amiens Sud, Amiens, France; Institut National de la Santé et de la Recherche Médicale U 872, France

## Abstract

Hepatitis C virus (HCV) is a leading cause of cirrhosis and liver cancer worldwide. We recently characterized for the first time the expression of Signaling Lymphocyte Activating Molecule 3 (SLAMF3) in human hepatocytes and here, we report that SLAMF3 interacts with the HCV viral protein E2 and is implicated in HCV entry process. We found a strong correlation between SLAMF3 expression level and hepatocyte susceptibility to HCV infection. The use of specific siRNAs to down-modulate SLAMF3 expression and SLAMF3-blocking antibodies both decreased the hepatocytes susceptibility to HCV infection. Moreover, SLAMF3 over-expression significantly increased susceptibility to HCV infection. Interestingly, experiments with peptides derived from each SLAMF3 domain showed that the first N-terminal extracellular domain is essential for interaction with HCV particles. Finally, we showed that recombinant HCV envelop protein E2 can bind SLAMF3 and that anti-SLAMF3 antibodies inhibited specifically this interaction. Overall, our results revealed that SLAMF3 plays a role during HCV entry, likely by enhancing entry of viral particle within hepatocytes.

## Introduction

The hepatitis C virus (HCV) particle entry into cells requires sequential interactions between viral proteins E1/E2 and several cellular factors. The recent development of functional models allowing the studies of the HCV life cycle, has prompted the identification of several cell surface proteins involved in HCV entry. Recent data suggest that HCV entry is a slow, complex, multistep process mechanism that depends on several cellular and environmental factors [1]. After initial attachment to the host cell (via GAGs and LDL-R, for example) [2], [3], HCV interacts with scavenger receptor class B type 1 (SR-BI), CD81 and claudin 1 (CLDN1) through its E1/E2 glycoprotein complexes [4], [5], [6], [7]. Other entry factors (such as occludin (OCLDN), receptor tyrosine kinases RTKs including the EGFR and EphA2 are thought to be involved at viral entry late stages [8], [9] by regulating CD81-CLDN1 coreceptor interactions and membrane fusion. Recently, NPC1L1 (a cholesterol sensing receptor expressed on apical surface of hepatocytes as well as enterocytes) [10] and transferin 1 [11] were identified as new HCV entry factor. The use of these all receptors leads to HCV internalisation by clathrin-mediated endocytosis [12], [13]. However, the viral entry process is far from being fully understood and may involve other membrane receptors and/or intracellular factors. Recently, we reported the expression by hepatocytes of only one single member from signaling lymphocytic activation molecule family (SLAM), the SLAMF3 (CD229) [14]. SLAM-family proteins have two or four extracellular immunoglobulin (Ig)-like domains, a transmembrane domain and an intracytoplasmic region containing tyrosine-based motifs. SLAMF3 has four extracellular Ig-like domains; domains 1 and 3 are very similar, as are domains 2 and 4 [15], [16]. It is noteworthy that SLAMF3 is associated with the µ-2 chain of the AP-2 adaptor complex and is the only SLAM family member that can be internalized by clathrin-mediated endocytosis [17], [18]. Until recently [13], SLAMF3 expression was documented for thymocytes, T cells, B cells, dendritic cells, macrophages and natural killer cells. During the formation of the immunological synapse between T cells and antigen-presenting B cells, SLAMF3 relocates at the edge of the contact area between the cells [19]. Nevertheless, SLAMF3's exact functions remain unclear. Interestingly, SLAMF1 acts as a measles virus co-receptor with CD46 [20]. As SLAMF3 is the only SLAMF family member that has been identified in hepatocytes, we examined it's role in HCV infection. We found that SLAMF3 blockade (with specific antibodies, domain-1 derived peptides and specific small interfering (siRNA) inhibits HCV entry into hepatocytes. Furthermore, over-expression of SLAMF3 enhanced the hepatocytes permissiveness to HCV. Taken together, our results suggest that hepatocyte SLAMF3 likely participates to viral entry as a cofactor.

## Material and Methods

### Cells, antibodies and peptides

Huh-7 cell line was a kind gift from Professor Gilles Duverlie (Virology Laboratory, Jules Verne Université Picardie, Amiens, France). The African Green Monkey kidney fibroblast COS-7 cell line was purchased from ATCC (Manassas, VA). All cell lines were maintained in Dulbecco's Modified Eagle's Medium (Life Technologies, Invitrogen, Saint Aubin, France) supplemented with 10% fetal calf serum (FCS) (PAA, Velizy-Villacoublay, France), 100 IU/mL penicillin, 100 µg/mL streptomycin and 2 mM L-glutamine. Healthy human primary hepatocytes PHH (Lonza, Basel, Switzerland) were maintained in phenol and serum-free HBCTM Basal Medium. HBCTM SingleQuotsKit containing 500 µL hEGF, 500 µL transferrin, 500 µL hydrocortisone, 10 mL bovine serum albumin (BSA), 500 µL ascorbic acid, 500 µL GA-1000 and 500 µL insulin was added to basal medium (Lonza, Basel, Switzerland). The following antibodies were used: a fluorescein isothiocyanate (FITC)-conjugated mouse mAb (HLy 9.1.25) and non-conjugated mouse mAbs (HLy 9.1.25, ZY-263 and 3H) against human SLAMF3 (AbD serotec, Colmar, France); non-conjugated and phycoerythrin (PE)-conjugated mAbs against human CD81 (JS-81)(BD Pharmingen, Le pont de Claix, France); unlabeled, PE-conjugated (Beckman Coulter, Villepinte, France) and FITC-conjugated isotype-matched mAbs (AbD serotec, Colmar, France), used as controls; polyclonal goat antibody against human SLAMF3 (K12) and actin (Santa Cruz Biotechnology, Heidelberg, Germany); FITC-conjugated rabbit anti-goat IgG, unconjugated and FITC-conjugated goat anti-rabbit IgG (Sigma-Aldrich, St Quentin Fallavier, France); non-conjugated rat-anti-E2 mAb (3/11, a kind gift from G. Duverlie, UFR Pharmcie, Amiens) and PE- conjugated goat anti-rat antibody (Jackson Immuno Research Laboratories). As control, the secondary anti-rat antibody was used alone. The goat anti-rabbit anti-E2 antibody (1/500, Ray Biotech), SLAMF3 peptides were synthesized by Genosphere (Paris, France) and registered under patent number PCT/EP2012/052398 (Inserm-Transfert-UPJV, Paris, France)

### Hepatocyte SLAMF3 blockade

Cells, from Huh-7 cell line and cultured primary human hepatocytes PHH, were treated with mAbs against CD81 (JS-81) or against the SLAMF3 receptor (3H, HLy9.1.25, ZY clones) or with the isotype controls for 1 h prior to incubation with HCVcc particles or HCV pseudoparticles HCVpp for 4 h. All preparations were azide free and low lipopolysaccharide LPS contents. Antibodies were used at 10 µg/mL as optimal concentration as determined by preliminary dose-response analyses. Cells were washed extensively and cultured in fresh medium for 72 h. For peptide competition assays, viruses were treated with an irrelevant control peptide (i.p., derived from the intracellular transcription factor STAT-5) or a SLAMF3-derived peptides (patent application PCT/EP2012/052398) prior incubation with cells. All peptides were used at 0.25 µM as optimal concentration as determined by preliminary dose-response analyses. No specific toxicity was obtained in the presence of SLAMF3 specific antibodies and derived peptides as evidenced by preliminary MTT toxicity test (bromure 3-(4,5-dimethylthiazol-2-yl)-2,5-diphenyl tetrazolium from Sigma-Aldrich, St Quentin Fallavier, France). Cells were lysed at 4 h and 72 h p.i. and the viral RNA load, number of focus-forming units (FFU) and E2 signal were evaluated.

### Hepatocyte SLAMF3 knock down by siRNA, transfections and primers

Three specific siRNAs (Ly9.1, Ly9.2 and Ly9.3, respectively ID114219 (Ly9-1, #1): 5′ CCAAGUGGAGUUACUCCCUtt-3′, ID212885 (Ly9-2, #2): 5′-CGUCCCAAAGAAAAUGUAAtt-3′, ID106775 (Ly9-3, #3): 5′-GGAAUUCACCCUGUUCGUCtt-3′) and the scrambled control (sc) siRNA were predesigned by Ambion and introduced into Huh-7 cells using the Silencer siRNA Transfection II Kit (Applied Biosystems, Ambion, Saint Aubin, France) according to the manufacturer's instructions. Briefly, 2×10^5^ cells were seeded in six-well plates and incubated for 50 h with 6 nM siRNA in the presence of 5 µL of NeoFX solution and with a final reaction volume of 100 µL of OptiMEM medium at 37°C. The transfection kit includes, positive control siRNA (targeting the GAPDH gene) and a negative control siRNA (a scrambled sequence that does not share homology with human, mouse or rat genome sequences). Gene expression was checked by mRNA quantification.

SLAMF3 cDNA from Huh-7 cells was cloned into a pBud vector. The following primers were used for PCR: forward 5′-GATCATCATGGTGGCACCAAAGAGTCACA-3′ and reverse 5′-GCAGCAGCTGCTTTTCCTTTCAGGTGAA-3′. After a purification step using the QIAquick gel extraction kit (Qiagen, Courtaboeuf, France), PCR products were cloned (using Topo TA cloning (Invitrogen Life Technologies)), sequenced and subcloned into the pBud CE4.1 vector using Kpn-1 and Xho-1 sites. For SLAMF3 over-expression in Huh-7 and COS-7 lines, cells (0.3×10^6^) were seeded into six-well dishes and transfected 24 h later with 0.8 µg of DNA by using the FuGENE HD Transfection Reagent Kit (Roche, Meylan, France) according to the manufacturer's instructions. After a 24 h incubation at 37°C, SLAMF3 transcripts and protein were quantified by QPCR, flow cytometry and Western blot analysis, respectively.

The following primers were used for QPCR to quatify HCV transcripts: forward primer (300 nM) HCV-S1 (nucleotides (nt): −215 to −197; 5′-TCCCGGGAGAGCCATAGTG-3′), 600 nM reverse primer HCV-AS2 (nt: −158 to −140; 5′-TCCAAGAAAGGACCCRGT-3′), and 250 nM TaqMan minor groove binding (MGB) probe labeled with 6-carboxyfluorescein (nt: −195 to −181; 5′-FAM-TCTGCGGAACCGGTG-MGB-3′) as previously described (Eurogentec, Angers, France) [21].

For SLAMF3 transcript quantification, the primers were as follows: forward 5′-TGG GAC TAA GAG CCT CTG GAA A-3′, reverse 5′-CCAGATGACGTTCTCAATCTC-3′ and an MGB probe with 6-FAM (5′-CCCCAACAGTGGTGTC-3′). The expression of the GAPDH mRNA was used as housekeeping endogenic control (human GAPDH VIC-MGB from Applied Biosystems) and to normalize the quantified mRNA.

The expression of SLAMF3 and CD81 at 48 h post-transfection was checked by mRNA quantification and western blot. For infection experiments, cells were infected at 48 h post-transfection of siRNA and then cultured for 72 h post-infection before infection evaluation.

For recombinant viral rE2 H77, the coding vector for E2 RGS-6HIS (H77, aa 384-664; kind gift from Dr. F.L. Cosset (INSERM U758), Lyon, France)) was transfected into HEK-293T cells and purified from culture supernatants after 72 hours of culture. The rE2 was used for an ELISA and dot blot assays as well as for binding tests as described elsewhere [22].

### HCVcc and HCVpp production, infection and measurements

The HCV strains JFH1 and H77/JFH1, HCVpps (1a-H77; 2a-HC-J6/CF; 3a-UKN3A1.28 and 4-UKN4.21.16) and a non-enveloped control were produced as described elsewhere [23]. As control, infections were performed by pseudoparticles harboring VSV-G envelope glycoproteins. For viral load quantification, viral RNA was extracted from cells and from culture supernatants using the RNeasy Qiagen Kit (Qiagen). In some experiments, the viral RNA viral load in collected supernatants were quantified, normalized for equal genome equivalents and used to infect native Huh-7 during an additional 72 h of infection. Viral RNA was extracted (using the QiAmpViral RNA Kit (Qiagen)) and analyzed on a 7900 HT Sequence Detection System running a TaqMan gene expression protocol (Applied Biosystems). After normalization against GAPDH, the results were expressed in arbitrary units (AU). Viral replication was assessed by FFU determination and by flow cytometry (3-11 anti-E2 and isotype-matched). Intracellular viral E2 protein staining was performed with the BD Cytofix/CytopermTM Plus kit (BD Biosciences) and cells were analyzed on a FACSCanto II system (Becton Dickinson). The data were then processed with FACSDiva software (Becton Dickinson) and FlowJo software (FlowJo, Ashland, USA). Data are expressed as the percentage of E2-positive cells. Hepatitis C virus pseudoparticles were produced and handled as described elsewhere [23], [24].

### Immunofluorescence analysis and FFU determination

For immunofluorescence staining, cells (10^6^/ml) were cultured on slides, washed in cold phosphate buffered saline PBS and fixed in 4% paraformaldehyde (PFA) for 10 min at room temperature (RT). Cells were washed and incubated with primary antibodies (1∶10) for 18 h at 4°C in a wet chamber. Next, the cells were washed in PBS-1% BSA-0.1% Tween20 and incubated with secondary antibody (1∶100) in PBS-BSA-Tween buffer for 30 min at RT. Following extensive washing in PBS/0.1% Tween20), slides were mounted with Vectashield and 4′,6′-diamidino-2-phénylindole DAPI for nuclear staining (Vector Laboratories, Burlingame, CA). The staining pattern was observed under a confocal microscopy (Zeiss LSM710) and analyzed with ImageJ software (FlowJo, Ashland, USA). For FFU determination, infected cells were detected by methanol-fixation and stained for E2 using the anti-E2 3/11 antibody and PE–conjugated anti–rat IgG. Infection was quantified by counting E2-positive cells forming colonies (FFU).

### Western blot analysis

Cells (10^6^ per assay) were lysed in Nonidet P40 (NP40) buffer (1% NP40, 50 mM Tris pH 7.5, 10% glycerol, 150 mM NaCL, 1 mM EDTA, 100 mM Na3VO6, 0.5 mM phenylmethanesulfonylfluoride PMSF, 5 mg/ml aprotinin, 5 mg/ml leupeptin and 2 mg/ml pepstatin) containing protease and phosphatase inhibitors (Roche, Meylan, France). Equal amounts of each protein sample were separated by electrophoresis on SDS-PAGE, blotted onto nitrocellulose membrane (Bio-Rad, Munich, Germany) and blotted with anti-SLAMF3 (K12) and anti-actin (C-11) antibodies. Blots were developed with the enhanced chemiluminescence (ECL) system (Amersham Pharmacia Biotech, Orsay, France).

### Dot-blot assays and ELISA

Recombinant E2 (0.5, 1, 2 and 5 µg) was immobilized on nitrocellulose membranes or in ELISA plate wells. After saturation in Tris buffered saline TBS/0.2% Tween/5% skimmed milk for 30 min at RT, the membranes or ELISA wells were incubated with goat anti-E2 antibody (1/500) overnight at 4°C. After several washes in TBS/0.2% Tween, a horse radish peroxidase HRP-conjugated secondary antibody anti-goat (1/10000) was added for 1 h at RT prior to detection with the ECL system (Amersham Pharmacia Biotech) for dot-blot assays and by substrate addition and optical density reading at 450 nm for ELISAs. To detect the direct rE2-SLAMF3 interaction, 5 mg of proteins extracted from Huh-7 and COS-7 cells were added to the rE2-coated nitrocellulose membrane for 4 h at RT. In some experiments, rE2 was tested at different concentrations (0.5, 1, 2 and 5 µg). Bovine serum albumin (5 mg) was used as control in similar conditions. The SLAMF3 or CD81 proteins having bound to the rE2 was detected by incubation with anti-SLAMF3 (K12, HLy9.1.25) and anti-CD81 (JS-81) antibodies, respectively, prior to detection with an HRP-conjugated anti-mouse antibody.

### Flow cytometry analysis

For surface staining, cells were treated with non-enzymatic cell dissociation solution (Sigma-Aldrich) for 10 min at 37°C, prior to washing in cold PBS/0.02% sodium azide/0.5% BSA. Cells (5×10^5^ per assay) were then incubated with conjugated mAbs or isotype-matched antibodies (5 µg/ml) for 20 min at 4°C. For indirect staining, cells were incubated for an additional 20 min at 4°C with secondary antibodies (1/100). After washing in PBS/0.02% sodium azide/0.5% BSA, cells were fixed with 1% PFA and stored in the dark at 4°C prior to analysis. The BD Cytofix/CytopermTM Plus kit (BD Biosciences) was used for intracellular E2 staining. After two washes with cold PBS, cells were incubated in 100 µL fixation/permeabilization solution for 20 min at 4°C and washed with BD Perm/WashTM Buffer 1× containing FCS and saponin. Next, the cells were incubated with the primary anti-E2 antibody in Perm/WashTM Buffer for 30 min at 4°C. After a wash with PBS, cells were incubated with the secondary anti-rat PE-conjugated antibody for 20 min.

### Statistical analysis

Results are expressed as the mean ± standard deviation (SD). Statistical analyses were performed with Prism graphpad software (Graphpad.com). Mean values of quantitative variables were compared in a Mann-Whitney rank-sum test. The threshold for statistical significance was set to p<0.05.

## Results

### SLAMF3-specific siRNAs inhibit HCV infection

**Figure 1:**
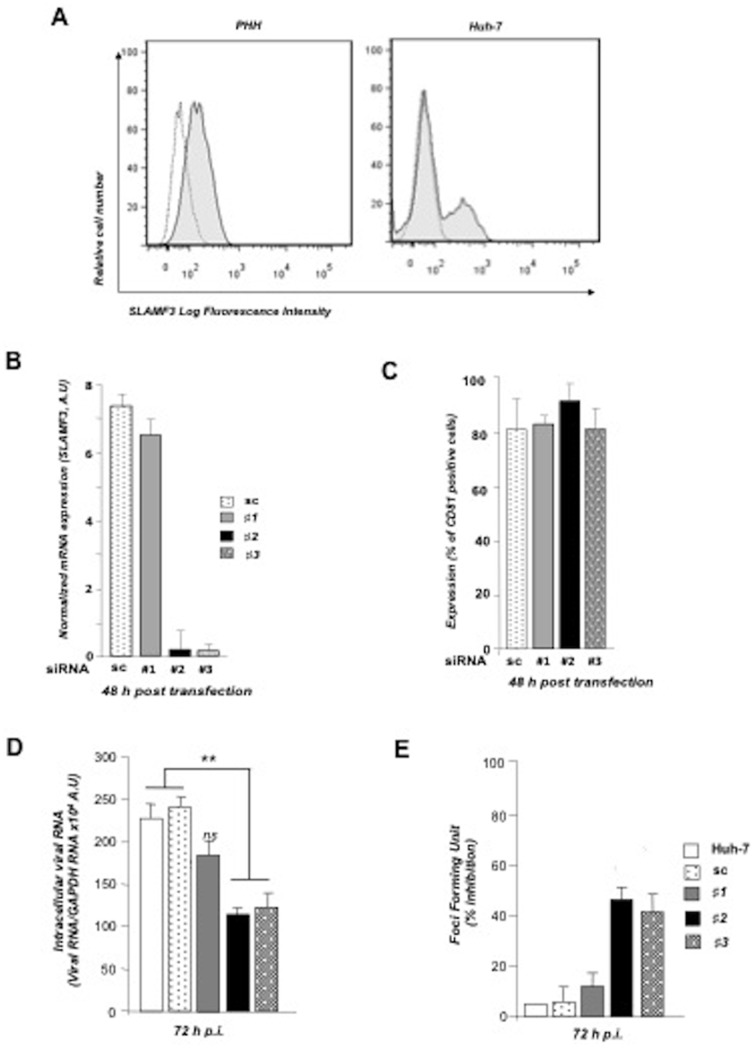
SLAMF3 blockade in human hepatocytes is associated with lower susceptibility to HCV. (A) SLAMF3 was stained in primary human hepatocytes (PHHs) and cells from the Huh-7 human hepatoma cell line with a specific antibody (HLy9.1.25 clone; grey) and an isotype-matched control (empty). One of four independent experiments is shown. Huh-7 cells were transfected with scrambled control (sc) siRNA or three specific siRNAs (#1, #2 and #3) targeting SLAMF3, prior to infection with HCVcc; siRNA efficiency was checked by quantifying SLAMF3 mRNA (B) and the CD81 expression level (C) by flow cytometry analysis at 48 h post-transfection. Results are presented as the mean ±SD (n = 3). Intracellular viral RNA was quantified at 72 h p.i. (D) and the infection was measured at 72 h p.i. by focus-forming units FFUs counting (E) (as inhibition percent; mean of three independent experiments; error bars: SD. **p<0.01);

Recently, we reported that the SLAMF3 is expressed by hepatocytes [13]. On average, 20% of Huh-7 cells and 50% of cultured primary hepatocytes PHH express SLAMF3 (Figure 1A). To evaluate the correlation between SLAMF3 expression and HCV susceptibility, we treated Huh-7 cells with SLAMF3-specific siRNA prior to the addition of JFH-1 cell-culture-derived infectious HCV virions (JFH-1 HCVcc) [25]. From the three SLAMF3 siRNA tested, two (#2 and #3) inhibited up to 90% fold SLAMF3 RNA expression, with the greatest effect observed at 48 h post-transfection (Figure 1B). No effect was obtained by Sc and #1 siRNA demonstrating the specificity of SLAMF3 inhibition. In addition, SLAMF3 protein expression is specifically inhibited as checked by western blot tests (Figure S1). As a control, we verified that the cell surface expression of CD81, one HCV receptor, was not affected by any siRNA (Figure 1C). We then infected these cells with JFH1 HCVcc. SLAMF3 siRNAs #2 and #3 significantly reduced HCV infection by a factor of two as demonstrated by viral replication (Figure 1D) and foci forming units inhibition in the whole cell population at 72 post-infection (p.i.) (Figure 1E).

### SLAMF3-specific antibodies decrease HCV entry

**Figure 2:**
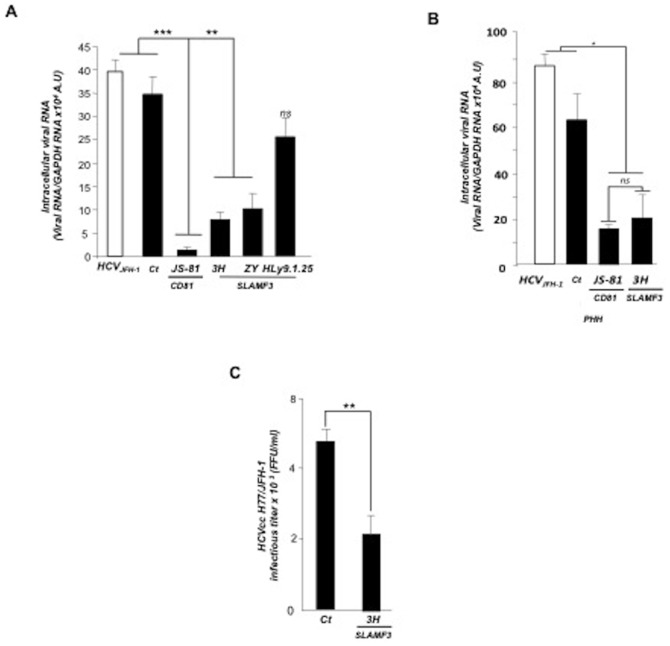
Antibodies against hepatocyte SLAMF3 reduce HCV infection. (A) Cells from the Huh-7 line were treated with anti-CD81 (JS-81) and/or anti-SLAMF3 (3H, ZY and HLy9.1.25 clones) mAbs prior to HCVcc infection (evaluated by quantification of intracellular vial RNA at 72 p.i.). (B) Cultured primary healthy human hepatocytes PHH were treated with anti-CD81 (JS-81) and/or anti-SLAMF3 (3H) mAbs prior to HCVcc infection (evaluated by quantification of intracellular viral RNA at 72 h p.i.). (Ct) refers to an isotype-matched control (in A and B), all neutralizing antibodies were used at 10 µg/ml; (C) The inhibitory effect of the 3H mAb on the number of FFUs after Huh-7 infection was checked at 72 h p.i. (mean of three independent experiments; error bars, SD * p<0.05, **p<0.01, ***p<0.005, ns: non-significant).

We then aimed to strength our observations by performing receptor blockade experiments with anti-SLAMF3 mAbs (3H, ZY and HLy9.1.25 clones; all of them bind to SLAMF3's extracellular domains). 3H and ZY mAbs significantly inhibited HCVcc replication (by 70% and 62%, respectively; p<0.01 for both, relative to control experiments) at 72 h p.i. (Figure 2A). No inhibition was obtained with HLy9.1.25 mAb, whereas the positive control, JS-81 anti CD81 antibody inhibited infection up to 90% (Figure 2A). Interestingly, the 3H and JS-81 mAbs showed a similar effect on HCV propagation in PHHs cultures by reducing infection by 70% (Figure 2B). Furthermore, the 3H mAb significantly reduced infection of Huh-7 by H77/JFH-1 HCVcc as measured by foci forming units (by up to 60%; p<0.01, relative to control experiments) (Figure 2C).

### SLAMF3 N-terminal extracellular domain is involved in the protein interaction with HCV

**Figure 3:**
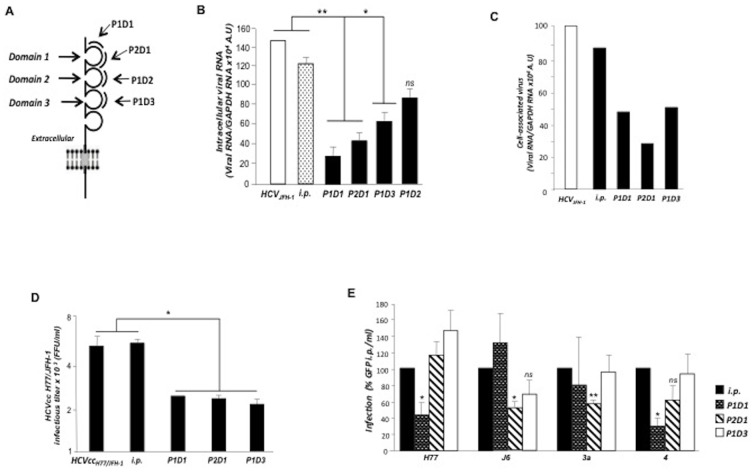
Hepatocyte SLAMF3-derived peptides inhibit HCV infection. For peptide competition assays, peptides derived from the first (P1D1 and P2D1), second (P1D2) and third (P1D3) extracellular domains of SLAMF3 and irrelevant peptide (i.p.) used at 0.25 µM were incubated with HCVcc prior to Huh-7 cells infection. (A) The diagram shows SLAMF3's topology and the position of SLAMF3 peptides used to prevent HCV attachment and infection; (B) the inhibitory effect was evaluated by quantification of intracellular viral RNA in infected Huh-7 cells at 72 h p.i. (mean of three experiments; error bars: SD; *p<0.05; **p<0.01, ns: non significant) and (C) in primary human hepatocytes (PHHs) presented as mean of one experiment; (D) Huh7 cells were challenged with HCVcc H77/JFH-1 and the inhibitory effect of SLAMF3-derived peptides was evaluated at 72 h p.i. and presented as FFU values (mean of three experiments; error bars: SD; *p<0.05); (E) The inhibitory effect of SLAMF3-derived peptides was tested on different pseudoparticles harboring envelope glycoproteins derived from HCV genotypes 1a-H77; 2a-HC-J6/CF; 3a-UKN3A1.28 and 4-UKN4.21.16 or from VSV (used as a control to normalize infection assays), (mean of three independent experiments; error bars: SD; *p<0.05, **p<0.01, ns, non-significant).

As SLAMF3 has four extracellular Ig-like domains showing similarities sequences (between domains 1/3 and 2/4) [15], [16], and to confirm the inhibitory effect of anti-SLAMF3 antibodies which specially recognize the first domain (3H, ZY and HLy9.1.25), We next tested the inhibitory effects of four 20-amino-acid synthetic peptides derived from SLAMF3's first, second and third extracellular domains (P1D1 and P2D1; P1D2; P1D3; Figure 3A) with both the HCVcc and HCV pseudoparticles (HCVpp) systems. Only peptides derived from the first and third domains inhibited HCVcc propagation and infection in Huh-7 cells (Figure 3B and D) and PHHs (Figure 3C). No significant inhibitory effect was obtained by P1D2. Using the HCVpp system, only peptides derived from domain 1 inhibited HCV infection (Figure 3 E). P1D1 significantly inhibited entry of HCVpps harboring a genotype 1a (H77) or genotype 4 derived envelope (p<0.05) whereas P2D1 inhibited entry of HCVpps harboring a genotype 2a (J6; p<0.05), or genotype 3a (p<0.01) derived envelope glycoproteins. Surprisingly, no significant inhibitory effects of HCVpp infections were observed in the presence of P1D3 (Figure 3 E).

### Increased SLAMF3 expression level enhances hepatocyte permissiveness to HCV infection

**Figure 4:**
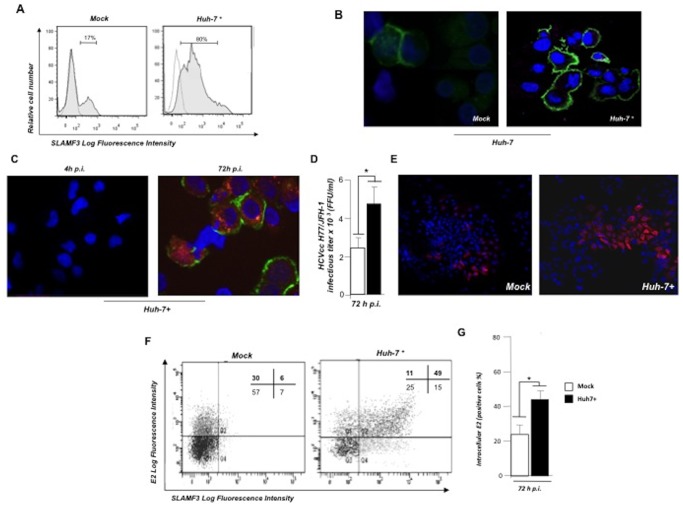
SLAMF3 over-expression increases hepatocyte permissiveness to HCV. Huh-7 cells were transfected with empty vector pBud (mock) or human SLAMF3 (to yield Huh-7+ cells) and incubated with HCVcc. (A) SLAMF3 expression was analyzed by flow cytometry (HLy9.1.25 clone) and (B) visualized by confocal microscopy in one out of three experiment. Unshaded histograms show the isotype-matched control (Co) and shaded histograms show SLAMF3 expression). (C, D, E) Infection was assessed at 4 and 72 h p.i. as the number of FFUs and (F and G) by flow cytometry analysis; (C) intracellular E2 (stained red) were measured at 4 h and 72 h p.i. Green staining corresponds to the SLAMF3 expression. One of three representatives independent experiments is shown. (D) The HCV permissiveness of Huh-7 mock and Huh-7+ cells was evaluated in terms of the number of FFUs based on intracellular E2 viral protein staining and (E) fluorescent microscopy analysis (mean of five experiments; error bars: SD; *p<0.05); Infection evaluation by flow cytometry analysis measuring the intracellular viral E2. (F) Infected cells (72 p.i.) were stained for surface expression of SLAMF3 (HLy9.1.25 clone) and permeabilized for intracellular E2 staining. The percentage of positive cells in each cell population is indicated in the corresponding quadrant (one of three representative experiments is shown) and (G) as a mean of three experiments (error bars: SD; *p<0.05).

To confirm the correlation between SLAMF3 expression levels and HCV susceptibility, we established Huh 7 cell lines that over-express SLAMF3 (Huh-7+). Flow cytometry analysis and immunofluorescence staining showed that 80% of the resulting Huh-7+ transiently expressed SLAMF3 (Figure 4A, B). We next infected these cells with JFH-1/HCVcc. Although no E2-positive Huh-7+ cells were detected at 4 h p.i., intracellular E2 were reproducibly detected at 72 hours p.i. (Figure 4C). The titer and proportion of infection in Huh7+ (E2+) cells was significantly higher than in for native Huh 7 cells (Figure 4C, D, E). A double staining of SLAMF3 and intracellular E2 expression measured by flow cytometry at 72 h p.i., demonstrated that 60% of the Huh-7+ cells were E2+ whereas only 36% of the native Huh 7 were E2+, suggesting that SLAMF3 over-expression increased hepatocytes susceptibility to HCV infection (Figure 4F, G).

### Viral particles released from SLAMF3-over-expressing cells are more infectious

**Figure 5:**
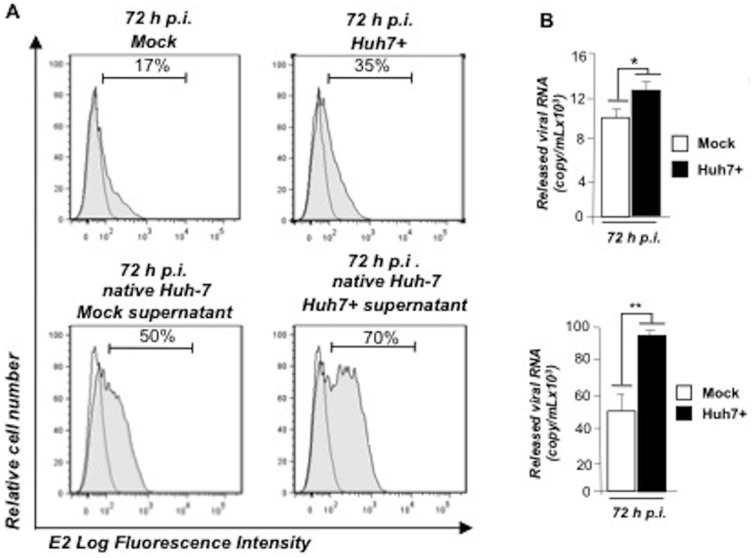
SLAMF3 over-expression increases the HCV infectivity. Mock and Huh7+ cells were challenged with HCVcc (A, top panel). (A) Supernatants were harvested at 72 h p.i., released viruses quantified by QPCR and equal viral loads from both supernatants were used to infect native Huh-7 cells (A bottom panel). (B) Viral replication was assessed by intracellular viral E2 protein expression measurement by flow cytometry and quantitation of released viral RNA at 72 h p.i. (mean of three independent experiments; error bars: SD *p<0.05, **p<0.01).

We then aimed to measure the infectivity of viral particles produced by SLAMF3 over-expressing Huh7 (Huh7+). After 72 hours of infection, Huh7 and Huh7+ cells were stained to measure percentage of E2 expression (Figure 5A, top panel) and HCV titers. In parallel, the released viral RNA were quantified using Q-RTPCR (Figure 5B, top panel) in order to infect native Huh7 cells with similar amount of HCVcc viruses produced either in Huh7 or Huh7+. Infectivity of viral particles in native Huh7 cells was then assessed 72 hours p.i. by measuring E2 expression by FACS analysis. Interestingly, infectious particles produced by Huh-7+ were able to infect more efficiently native Huh-7 than viral particles produced by native Huh-7 cells (Figure 5A bottom panel). In agreement with the increased infection, the released viral load was higher in native Huh-7 cells cultures infected by Huh-7+ standardized supernatants than those of Huh-7 mock cultures (Figure 5 B).

### Hepatocyte SLAMF3 is involved in E2 binding to hepatocytes and interacts with soluble viral E2

**Figure 6:**
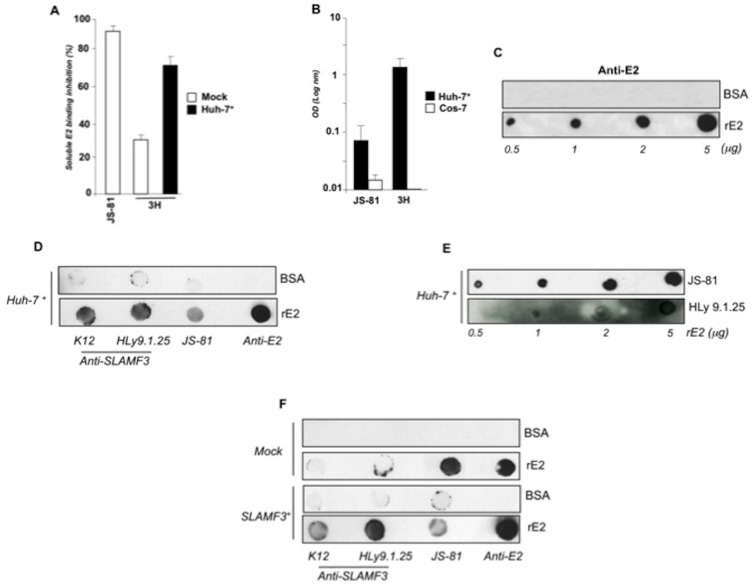
Recombinant viral E2 (rE2) interacts with SLAMF3. (A) Mock and Huh-7+ cells were fixed (in 4% PFA for 10 min) prior to incubation with rE2 for 1 h at 4°C. To measure binding inhibition, cells were incubated with anti-CD81 (JS-81), anti-SLAMF3 (3H) or isotypes for 30 min prior to incubation with rE2 and detection of its binding with anti-E2 antibody by flow cytometry (the mean of two experiments; error bars: SD); (B) Extracted proteins from Huh-7+ and COS-7 were incubated with coated rE2 on an ELISA plate before extensive washing and specific detection of bound CD81 (JS-81) and SLAMF3 (3H) proteins. The optical density (OD) was read at 580 nm and is presented as the mean of two experiments; error bars: SD; (C) Recombinant soluble HCV E2 (rE2) and bovine serum albumin (BSA, used as a control) were coated on nitrocellulose membranes (at the indicated concentrations) prior to incubation with 3/11 E2-specific antibody; (D) rE2 and BSA (5 µg) were coated on nitrocellulose membranes and then incubated with proteins from Huh-7+ cells. Spots were detected using anti-E2 (positive control), anti-SLAMF3 (K12 and HLy9.1.25) and anti-CD81 (JS-81) antibodies; (E) CD81 and SLAMF3 bound to rE2 used at the indicated concentrations were incubated with anti-CD81 (JS-81) and anti-SLAMF3 (HLy9.1.25) antibodies; (F) proteins from native COS-7 cells (top; mock) or SLAMF3-over-expressing COS-7 cells (bottom) were incubated with rE2 and BSA coated on nitrocellulose membranes prior to CD81 and SLAMF3 detection with specific antibodies. One representative experiment from three independent experiments is presented in A, D, E and F.

Next, we used soluble rE2 (1a genotype, H77 strain, 383-663) in a series of binding inhibition assays. Assessment of rE2 binding to Huh-7 and Huh7+ following incubation of these cells lines with JS81 or with three different anti-SLAMF3 mAbs (HLy9.1.25, ZY and 3H clones) showed that anti-SLAMF3 3H mAb was able to prevent rE2 binding to Huh-7 (by up to 30%). Furthermore, binding inhibition reached 70% for Huh-7+ cells. In control experiments, JS-81 mAb inhibited rE2 binding by up to 90% (Figure 6A). No inhibitory effects were observed in the presence of HLy9.1.25 and ZY (data not shown). In order to determine whether SLAMF3 interacts directly with rE2, we incubated membrane proteins extracted from Huh-7+ cells with rE2 immobilized in plastic wells for ELISA or on nitrocellulose membranes for dot blot assays. Protein extracts from the green monkey cell line COS-7 (which does not express SLAMF3) were used as negative controls. The ELISA results showed that rE2 was able to bind both CD81 and SLAMF3 (detected by JS-81 and 3H, respectively) (Figure 6B). The specificity of the 3/11 anti-E2 mAb to recognize coated rE2 in a dot blot test was verified (Figure 6C). For SLAMF3 detection on nitrocellulose membranes, we used the HLy9.1.25 and ZY clones which are more suitable for dot-blot assays. Dot-blot tests confirmed that the interaction between rE2 and SLAMF3 could be detected by different anti-SLAMF3 antibodies (Figure 6D) and occurs in a dose-dependent manner similarly to rE2-CD81 interaction (Figure 6 E). rE2-SLAMF3 interaction is specific as a rE2-SLAMF3 interaction was detected only in COS-7 ectopically expressing SLAMF3, but not in extracts from native (non-transfected) COS-7 cells (Figure 6F).

## Discussion

Here, we demonstrated that the SLAMF3 expressed by human hepatocytes enhances HCV entry. Several studies have shown that CD81 and SR-BI interact with soluble E2 and are major receptors for HCV entry [5], [26]–[28]. Recently, it was showed that E1E2 complexes can interacts with the first extracellular loop of Claudin-1, whereas soluble E2 did not [7]. In contrast, direct interactions between viral proteins and the tight junction proteins OCLDN, identified as late-stage HCV receptors, have not yet been described [6]–[8]. Recently, we show that SLAMF3 is highly expressed by healthy human hepatocytes whereas its expression is decreased in HCC cells [14]. Here, we showed that anti-SLAMF3 mAb (3H) decreased rE2 binding to Huh-7+ cells by 70% compared with 30% in native Huh-7 cells. Interestingly, the 3H mAb, that recognized the first extracellular domain of SLAMF3, inhibited HCVpp and HCVcc infections. Other mAb, such as ZY, inhibited HCVcc infection to a lesser extent. Thus, it is likely that the SLAMF3 mAbs recognize different SLAMF3 epitopes involved at different degree in SLAMF3-related HCVcc infection. Competition experiments with SLAMF3-derived peptides revealed that domain-1-derived peptides (P1D1 and P2D1) inhibited HCVpp (envelope 1a, 2a, 3a and 4) and HCVcc infection. Our results suggest that the most exposed first extracellular domain of SLAMF3 was implicated in the interaction between viral particles and SLAMF3. Taken together, the inhibitory effects of mAb directed against the SLAMF3 domain 1 combined to the inhibitory effect of domain 1-derived peptide suggest that this domain is potentially involved in virus binding and HCV infection. The HCV particles incorporate two envelope glycoproteins E1E2 onto their surface. We assessed whether soluble rE2 directly interacts with SLAMF3 extracted from hepatocytes. Interestingly, the SLAMF3 protein extracted from hepatocytes bound the coated rE2 in a similar way to CD81 demonstrating a possible direct interaction between SLAMF3 and the E2 viral protein. This interaction was specific as demonstrated by the ability of the 3H mAb to inhibit rE2 binding on Huh-7+ cells. Importantly, no rE2-SLAMF3 interaction was observed using COS-7 cell protein extracts (that do not express SLAMF3) whereas a rE2-SLAMF3 interaction was observed with COS-7 protein extracts after ectopic expression of SLAMF3. This result suggests that the observed rE2-SLAMF3 interaction was relevant and specific. Taken together, our results highlight a potent interaction between viral E2 and SLAMF3 proteins. Additional tests will be necessary to evaluate the affinity of this interaction and its genotype-dependence.

In summary, we demonstrated that the hepatocyte SLAMF3 is potentially involved in HCV infection through interaction with E2 glycoprotein and enhancement of virus entry. How and when this enhancement occurs during the different stages of HCV entry remain to be elucidated. SLAMF3 seems to be an attachment factor as seen by virus or rE2 binding experiments. However, we cannot exclude that HCVcc binding may be achieved via lipoprotein components of the HCV particle. Additional experiments need to be designed. In addition, since SLAMF3 is the only SLAM family member with the potential to be internalized through a clathrin-dependent pathway [16], [17], SLAMF3 could act during later stages of virus entry and enhances internalization of viral particles with CD81/Claudin-1 complexes. This question remains to be explored. SLAMF3 may therefore be a HCV entry cofactor that plays a role in HCV infection at the level of glycoprotein-mediated entry and possibly in HCV particle internalization, in a similar extent to the transferrin receptor 1 that were recently described [11]. All these studies will help to define more precisely the sequence of events initiated by attachment of the virus to its host cell end leading to the release of the viral genome into the cytoplasm. To achieve this clear picture, the identification of all cell entry factors is crucial. HCV entry is clearly an unique and complex mechanism. Then, the individual role of each attachment molecule, of each receptor involved in major E1E2 conformational changes and of each cofactor important for virus internalization will need to be characterized and understood at the molecular level.

## Supporting Information

Figure S1 **Huh-7 cells were transfected with scrambled control (sc) siRNA or three specific siRNAs (#1, #2 and #3) targeting SLAMF3.** Proteins were extracted and analyzed by western blot using polyclonal anti-SLAMF3 (K12 clone). One of two independent experiments was presented.
(TIFF)
