# Supplementary figures and images for: The Expression of the Hepatocyte SLAMF3 (CD229) Receptor Enhances the Hepatitis C Virus Infection
Source: PLoS One. 2014 Jun 13;9(6):e99601. doi: 10.1371/journal.pone.0099601 (PMC4057114; doi:10.1371/journal.pone.0099601)

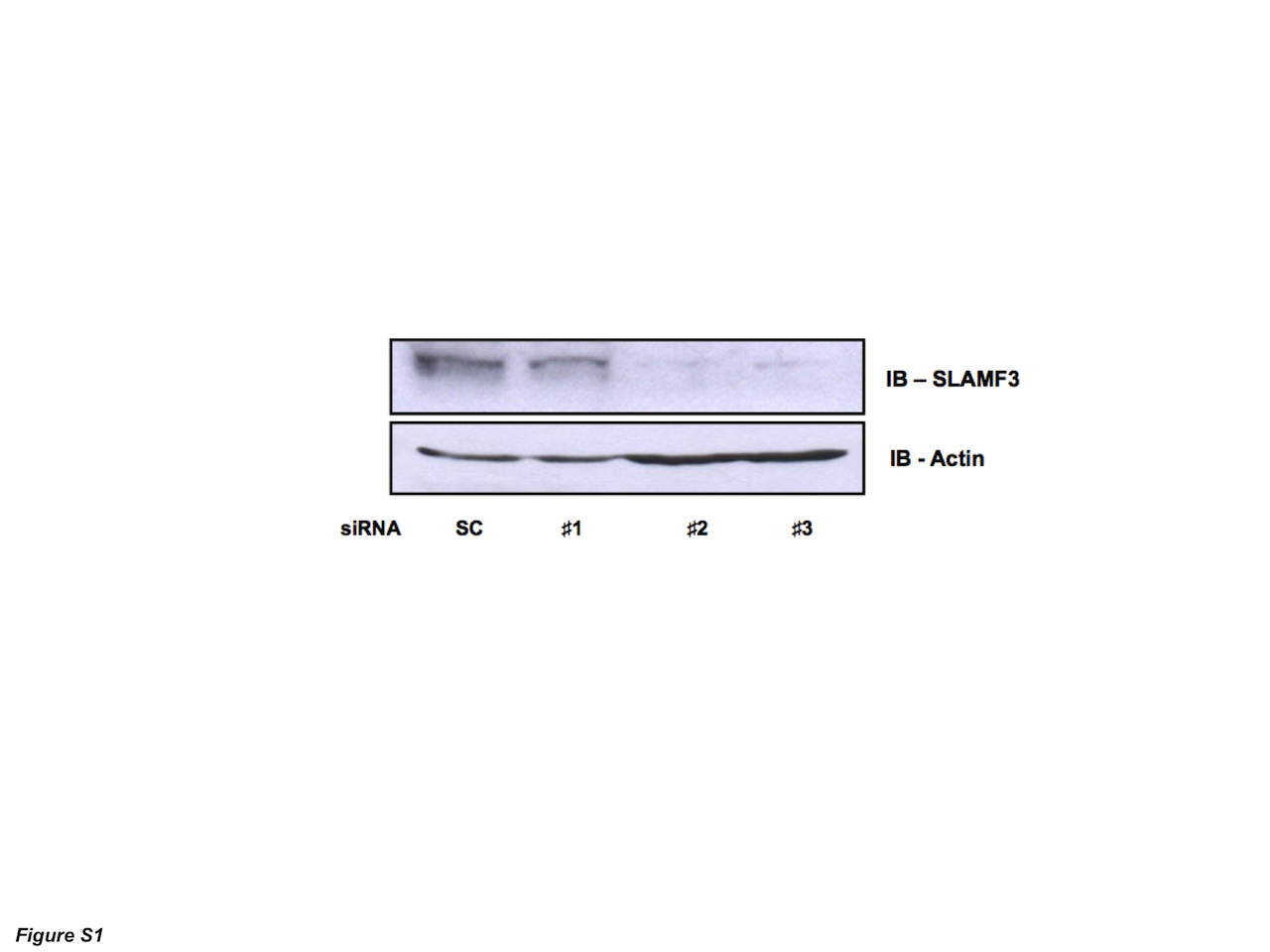

Supplement: Figure S1 — Huh-7 cells were transfected with scrambled control (sc) siRNA or three specific siRNAs (#1, #2 and #3) targeting SLAMF3. Proteins were extracted and analyzed by western blot using polyclonal anti-SLAMF3 (K12 clone). One of two independent experiments was presented. (TIFF) [file pone.0099601.s001.tiff]
